# Investigation of the mechanical work during ultrasonic fatigue loading using pulsed time-resolved X-ray diffraction

**DOI:** 10.1107/S1600577523008767

**Published:** 2024-01-01

**Authors:** Vincent Jacquemain, Christophe Cheuleu, Nicolas Ranc, Olivier Castelnau, Vincent Michel, Doriana Vinci, Véronique Favier, Cristian Mocuta, Dominique Thiaudiere

**Affiliations:** a PIMM, Arts et Metiers Institute of Technology, CNRS, CNAM, HESAM University, 151 Boulevard de l’Hopital, Paris, France; b European XFEL GmbH, Holzkoppel 4, 22869 Schenefeld, Germany; c Synchrotron SOLEIL, L’Orme des Merisiers, Départamentale 128, 91190 Saint-Aubin, France; Advanced Photon Source, USA

**Keywords:** X-ray diffraction, ultrasonic fatigue machine, very high cycle fatigue, pump–probe method, pulsed X-ray source

## Abstract

In the energy production and transportation industries, numerous metallic structures may be subjected to at least several billions of cycles, *i.e.* loaded in the very high cycle fatigue domain (VHCF). Therefore, to design structures in the VHCF domain, a reliable methodology is necessary. One useful quantity to characterize plastic activity at the microscopic scale and fatigue damage evolution is the mechanical work supplied to a material. However, the estimation of this mechanical work in a metal during ultrasonic fatigue tests remains challenging. This paper aims to present an innovative methodology to quantify this. An experimental procedure was developed to estimate the mechanical work from stress and total strain evolution measurements during one loading cycle with a time accuracy of about 50 ns. This was achieved by conducting time-resolved X-ray diffraction coupled to strain gauge measurements at a synchrotron facility working in pulsed mode (single-bunch mode).

## Abstract 2

The development of a time-resolved X-ray diffraction technique using synchrotron radiation in pulsed mode to estimate the stress and the mechanical work during an ultrasonic fatigue test is presented.

## 1. Introduction

For more than 150 years it has been known that mechanical structures can break under repeated loading well below the yield stress or rupture stress for monotonic loading. This phenomenon is called fatigue failure. Fatigue failure is of great importance in materials science today because a major part of mechanical systems in the transport and energy production industries are designed to resist fatigue loading. Fatigue mechanisms often affect metallic parts in heavily loaded components that are critical to the reliability and the security of the structure to which they belong, such as the rotating parts of thermal engines in the car industry and turbine blades in turbojets and power plants. There is thus a great need for reliable fatigue design methods.

Current design methods for a structure loaded under fatigue make use of standards (ISO-12107, 2012 ▸) that are based upon the concept of the fatigue limit introduced by Wöhler in the 1860s (Wöhler, 1870 ▸). For the standards it was recommended to carry out tests on hydraulic tensile machines and to determine a relationship between the stress amplitude and the number of cycles needed to break the specimen. The obtained curve is known as the stress number of cycles curve (SN curve) and it characterizes the fatigue behaviour of the material. For a high number of cycles (higher than 10^7^), the standards suppose that the SN curve presents a horizontal asymptote called the fatigue limit. For stress amplitudes lower than this fatigue limit or for numbers of cycles higher than 10^7^, the tested specimen or the structure should never break. This is referred to as unlimited endurance or infinite lifespan.

Plotting a SN curve thus requires carrying out fatigue tests for various stress amplitudes. Taking into account the probabilistic nature of fatigue failure, it is necessary to conduct a minimum of 25 tests to design a SN curve. With a traditional hydraulic machine working at a frequency of 30 Hz it takes more than one month to plot a single SN curve and to thus completely characterize the fatigue behaviour of a material in the high-cycle fatigue domain (HCF).

For cost and environmental reasons, it is often necessary to increase the lifespan of structures and to reduce the number of maintenance phases. This leads thus to an increase in the number of cycles applied to the structure. In the automotive industry the valve spring in a thermal engine can be submitted to 10^8^ to 10^9^ mechanical cycles over the lifespan of the engine. In the aeronautic industry, low-amplitude cyclic loadings can be superimposed on higher-amplitude cyclic loadings associated with the takeoff and landing of a plane. These low-amplitude cyclic loadings are associated with high-frequency vibration, and the number of cycles can reach 10^10^ to 10^11^. These examples illustrate the need to characterize the fatigue mechanisms in the domain of the gigacycle fatigue regime, also called the very-high-cycle fatigue (VHCF) domain, corresponding to more than 10^7^ cycles. In spite of these obvious examples, industry has almost never considered fatigue design beyond 10^7^ cycles because of difficulties related to the test duration. Generally, in accordance with standards, the concept of the fatigue limit is supposed to be valid and extrapolated to a billion or more cycles but some studies show that this assumption is not always valid (Bathias, 1999 ▸; Nishijima & Kanazawa, 1999 ▸; Shiozawa & Lu, 2002 ▸).

To overcome the limitations of hydraulic test machines, reduce the test durations and explore the gigacycle fatigue domain, ultrasonic fatigue machines working at a frequency of 20 kHz have been developed over the past three decades (Wu *et al.*, 1994 ▸). With this type of machine, it becomes possible to establish SN curves up to 10^9^ cycles in a reasonable time, typically a few days.

In parallel to the development of ultrasonic fatigue machines, new methods for the rapid determination of the fatigue behaviour, based on the measurement of the specimen self-heating, have been developed. These methods involve measuring the temperature increase during cyclic loading. Indeed, self-heating originates from internal heat sources (visco-elastic behaviour, dislocation motion, friction of crack surfaces, *etc.*) that are activated during the fatigue test. A steady-state temperature associated with the current stress amplitude is generally reached after about 10^4^ cycles. Sequences of 10^4^ cycles with increasing stress amplitude are then applied to the specimen to obtain the temperature increment evolution versus stress amplitude. The conventional fatigue limit is then given in an empirical way using a threshold from which the temperature increases rapidly with the stress amplitude (Luong, 1998 ▸). This methodology is particularly easy to implement because high-performance experimental devices like infrared cameras are commercially available for temperature measurement. Moreover, to overcome the effects of geometry or thermal boundary conditions imposed by the environment, these methods have been improved by estimating, from the evolution of the temperature field, the mean energy dissipated in the material per unit volume and time, called intrinsic dissipation (Chrysochoos *et al.*, 2008 ▸). The fatigue behaviour is then characterized by an evolution of this intrinsic dissipation as a function of the stress amplitude. This methodology was developed initially using standard hydraulic machines (Boulanger *et al.*, 2004 ▸) and more recently extended to ultrasonic fatigue machines (Blanche *et al.*, 2015 ▸).

Even if the method based on the self-heating method provides interesting information on the fatigue properties, the intrinsic dissipation remains difficult to interpret physically in terms of the level of damage of the material (Favier *et al.*, 2016 ▸). A recent alternative was rather to estimate the energy stored by the material, as this quantity is directly related to the evolution of the dislocation density and the formation of ladder-like dislocation structures resulting from the accumulation of plastic deformation (Mughrabi, 2006 ▸, 2015 ▸). Dis­locations, as other crystal lattice defects, distort the crystal lattice and thus create a field of elastic strain (and associated stress) from which the stored energy can be calculated. The stored energy remains, however, more difficult to evaluate than the intrinsic dissipation, especially when the loading frequencies are high because it is necessary to estimate simultaneously the dissipated energy and the mechanical work brought by the machine to the material. Then, the stored energy can be quantified using the principles of thermodynamics (Chrysochoos *et al.*, 2008 ▸) through the following equation,

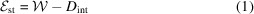

where 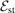 , 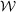 and *D*_int_ are, respectively, the stored energy, the mechanical work and the dissipated energy during one cycle. The estimation of the mechanical work remains nowadays very challenging. It consists of an intermediate measurement which could be used to estimate the stored energy and requires measurement of stress and total strain rate evolutions during one cycle. The total strain rate is easily measured using a strain gauge and the stress can be estimated by X-ray diffraction as detailed in Section 2. An experimental device has already been proposed by our research team to make these measurements during ultrasonic fatigue tests (Jacquemain *et al.*, 2021 ▸). However, owing to the low damping of metals, the opening of the hysteresis loop in a stress–strain diagram remains very small and thus it is necessary to be able to detect tiny time shifts between stress and strain that are largely smaller compared with the loading period. If an ultrasonic fatigue machine is used to apply the cyclic loading, the expected time shift is about a few tens of nanoseconds. To measure experimentally this time shift, the use of a synchrotron pulsed X-ray source is necessary. Pump–probe techniques are commonly employed to achieve nanosecond time resolution (Rehn *et al.*, 1990 ▸; Koliyadu *et al.*, 2022 ▸). Very often, a laser is used as pump and the specimen is probed by X-rays in many scientific domains such as biology (Berera *et al.*, 2009 ▸), chemistry (Britz *et al.*, 2016 ▸), physics (Laulhé *et al.*, 2012 ▸; Silly *et al.*, 2017 ▸) and materials science (Gonzalez Vallejo, 2019 ▸). Here, we propose to use a piezo-electric converter as the pump to cyclically load fatigue specimens.

The objective of this paper is therefore to propose an experimental method sufficiently resolved in time to correctly estimate *in situ* the stress by X-ray diffraction and ultimately the mechanical work supplied during an ultrasonic fatigue test. The first part of the paper will be dedicated to the presentation of the experimental device with a particular focus on the synchronization technique and the time resolution characterization. The second part will concern the presentation of the results and their analysis.

## 2. Experimental procedure

### 2.1. Ultrasonic fatigue machine and specimen

**Figure 1:**
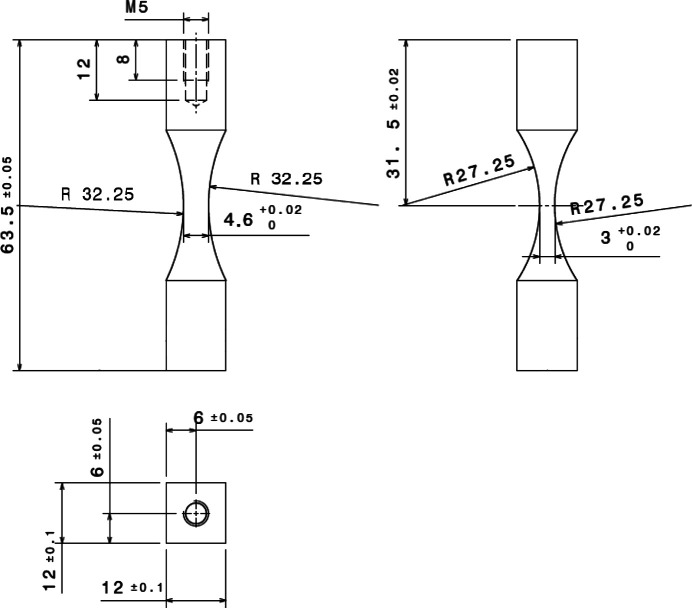
Geometry of the ultrasonic fatigue specimen (pearlitic steel).

The experimental device used in this study is composed of an ultrasonic fatigue machine to load cyclically a specimen at a frequency close to 20 kHz. The ultrasonic fatigue machine consists of a piezo-electric converter (CR20 model from Branson), and a booster and a horn with amplification coefficients of 1.5 and 2.5, respectively. A specimen with a rectangular central cross section of 4.6 mm by 3 mm is fixed on the horn and vibrates at its first longitudinal vibration mode close to 20 kHz. During the loading both the converter and specimen are refreshed by air flow to limit the temperature increase of the specimen and the thermal stability of the converter.The material of the specimen is a biphasic pearlitic steel (C70) with ferrite and cementite phases. The geometry of the specimen is determined using a modal calculation based on the finite-element code *Abaqus* and is given in Fig. 1 ▸. More details on the design methodology are given by Jacquemain *et al.* (2021 ▸). It is to be noted that the fatigue limit was measured after 10^9^ cycles for this material and was found to lie close to 400 MPa.

### 2.2. Estimation of the mechanical work supplied to the material during one cycle

#### 2.2.1. Mechanical work estimation

To calculate the mechanical work supplied to the material during one cycle, the time evolutions of the longitudinal stress σ(*t*) and the total longitudinal strain ɛ(*t*) (taking into accound both elastic and plastic strains) must be estimated during one cycle. The supplied mechanical work can then be quantified using the equation below,

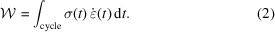

Assuming that the evolutions of stress and total strain remain close to sinusoidal evolutions [ 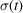 = 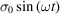 and 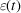 = 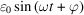 with σ_0_ and ɛ_0_ the stress and total strain amplitudes, respectively, and φ the phase shift of the total strain with respect to the stress], equation (1) can be simplified to

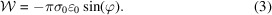

This assumption is generally valid in the context of the low stress amplitudes associated with the VHCF domain. The estimation of the supplied mechanical work to the material then requires the measurement of the stress and the total strain amplitudes as well as the time shift between these two quantities.

#### 2.2.2. Estimation of the total longitudinal strain

The total longitudinal strain of the specimen, which is the sum of both elastic and plastic strains, is measured using strain gauges (KFGS-1N-120-C1-11 from Kyowa company) glued to both sides of the specimen and placed in a full Wheatstone bridge to measure the mean value of the loaded gauges to remove the effect of room-temperature variation during a long test. In addition, the model of strain gauge used in this study is thermally compensated to remove the effects of dilatation on the strain estimate. The size of the gauges grid is 1 mm length by 0.65 mm width. The strain gauge’s signal is amplified using a CDV 900A Kyowa conditioner.

#### 2.2.3. Estimation of the stress

**Figure 2:**
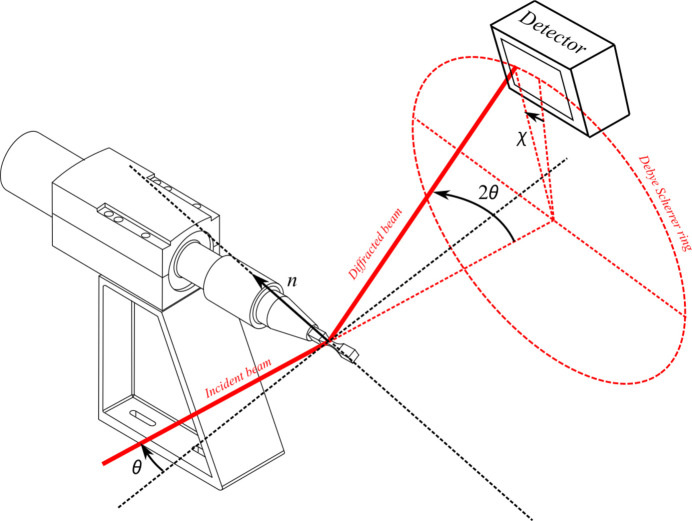
Schematic view of the experimental device for the stress measurement by X-ray diffraction.

For the stress estimation byX-ray diffraction, an ultrasonic fatigue machine is installed on the diffractometer of the DiffAbs beamline at Synchrotron SOLEIL (see https://www.synchrotron-soleil.fr/en/beamlines/diffabs). The incident X-ray beam is monochromatic and its energy is set at 18 keV. It is positioned in the centre of the useful part of the specimen and has a footprint on the sample of 200 µm × 500 µm [full width at half-maximum (FWHM), vertical × horizontal]. The Debye Scherrer ring originating from the {110} lattice planes of the ferrite phase is observed in reflection in a θ–2θ configuration with a hybrid pixel XPAD detector with a matrix of 560 × 240 pixels (pixel size: 130 µm) distributed over seven modules (Pangaud *et al.*, 2007 ▸; Medjoubi *et al.*, 2010 ▸; Le Bourlot *et al.*, 2012 ▸). The distance between the specimen surface and the detector is about 710 mm. A scheme of the experimental configuration is given in Fig. 2 ▸.

**Figure 3:**
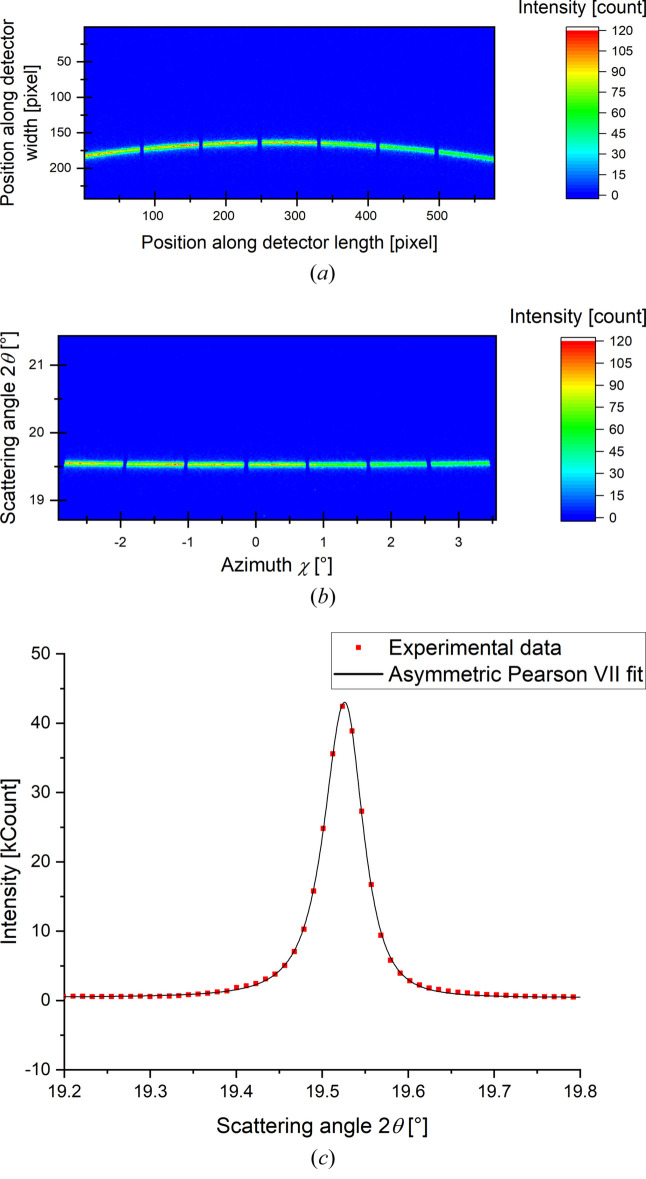
Analysis methodology of diffraction patterns. (*a*) X-ray detector (raw) image of the {110} Debye Scherrer ring. (*b*) Masking of hot, dead and inter-modules double pixels of the image, transformation of the image in the azimuth and scattering angle coordinate system [for further details, see Ors *et al.* (2019 ▸)]. (*c*) Azimuthal integration and fit of the diffraction peak with an asymmetric Pearson VII function.

The analysis of diffraction patterns is caried out with a method similar to that presented by Ors *et al.* (2019 ▸). The different steps, illustrated in Fig. 3 ▸, are (i) a transformation of the image obtained with the X-ray detector in the scattering angle and azimuth angle coordinate system, (ii) an azimuthal integration using the *AzimuthalIntegrator* class of the *pyFAI* package (Ashiotis *et al.*, 2015 ▸) and (iii) a fit with an asymmetric Pearson VII function,

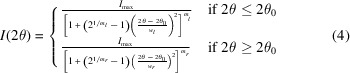

where *I*_max_, 2θ_0_, *w*_*l*_, *w*_*r*_, *m*_*l*_ and *m*_*r*_ are the fit parameters representing, respectively, the height of the peak, the peak position, and parameters related to the widths (*w*) and shapes (*m*) of the two branches. To take the background into account, a linear function is added to the Pearson VII function. The position of the diffraction peak is then estimated from the 2θ_0_ parameter. The displacement of the {110} peak is converted to the macroscopic normal stress along the specimen axis, noted σ, using a scale transition model (elastic self-consistent scheme) to compute the X-ray elastic constant (Noyan & Cohen, 1987 ▸; Vermeulen, 2001 ▸; Faurie *et al.*, 2009 ▸; Purushottam Raj Purohit *et al.*, 2021 ▸),

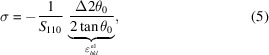

with 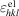 the elastic lattice strain, 2θ_0_ the reference angular position of the diffraction peak without applied loading, Δ2θ_0_ the variation of the diffraction peak angular position during the loading and *S*_110_ the X-ray elastic constant for the {110} diffraction planes family. Jacquemain *et al.* (2021 ▸) give an estimation of *S*_110_ of about −1.32 × 10^−6^ MPa^−1^ for a random crystallographic texture and an equiaxed mean grain shape.

### 2.3. Synchronization and data acquisition

#### 2.3.1. Synchronization of the ultrasonic machine

Metallic materials loaded in the VHCF domain involve low dissipated energy and can generate a very small time shift between total strain and stress which can go down to 50 ns. An estimation of the mechanical work carried out on the specimen using equation (2) is therefore required to reach this temporal resolution. The synchronization and measurement methods developed previously in the literature (Ors *et al.*, 2019 ▸) to estimate the stress by X-ray diffraction during an ultrasonic fatigue test only allow temporal resolutions of the order of 1 µs or slightly lower to be achieved. This limitation essentially comes from the minimum aperture time of the XPAD X-ray detector.

To improve our experimental device and reach the targeted time resolution, the pulsed X-ray source available on the DiffAbs beamline of Synchrotron SOLEIL in single-bunch mode is used (Couprie *et al.*, 2013 ▸; Nadolski *et al.*, 2018 ▸). This mode allows an X-ray pulse with a duration of 91 ps FWHM, and the associated current in the storage ring is about 16 mA. Synchronization of our devices (ultrasonic fatigue machine, strain and stress measurement) is done from the radio-frequency (RF) cavity clock whose frequency is about 352.197 MHz. This frequency can vary by about ±2 kHz to compensate for changes on the storage ring before an experimental campaign and by about ±25 Hz for orbit corrections during a single experimental campaign. The clock of the storage ring is synchronized with the clock of the RF cavity and its frequency is equal to the frequency of the RF cavity divided by 416, *i.e.* 846.6 kHz (corresponding to the maximum number of bunches that the ring can contain). This frequency enables a revolution time of the electrons bunch of about 1.1812 µs to be calculated, which is equal to the time between two X-ray pulses. The synchronization of the electronic devices with the storage ring clock is performed via a TimBeL synchronization board (Ricaud *et al.*, 2011 ▸) available on the DiffAbs beamline. The rising edge of the TimBeL signal will be considered as the time origin for all measurements. The jitter on the synchronization signal is about 4 ps RMS.

**Figure 4:**
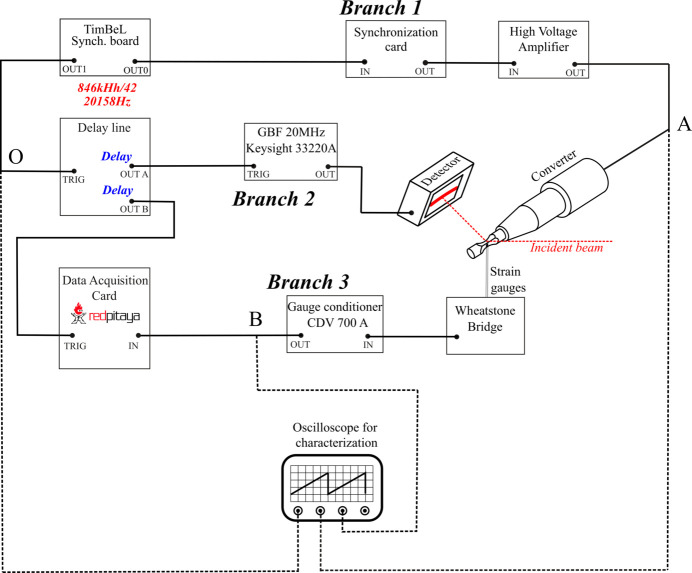
Graphical representation of the synchronization device.

The control of the fatigue machine and its synchronization with the master clock of the synchrotron storage ring are ensured by a synchronization electronic card and a high-voltage amplifier (branch 1 in Fig. 4 ▸). The synchronization card allows a sinusoidal voltage to be generated at a frequency equal to the frequency delivered by the TimBeL board (∼846.6 kHz) divided by 42 namely, 20158 Hz. The variation of this frequency due to the RF cavity compensation during an experimental campaign is about ±0.005 Hz. The factor 42 is chosen to obtain a frequency as close as possible to the nominal working frequency of the ultrasonic fatigue machine. The synchronization card ensures a constant delay (close to zero) between the synchronization signal of the TimBeL and the generated sinusoidal signal, with a time accuracy of the order of the jitter introduced by the synchronization card. The voltage of the synchronized sinusoidal signal is then amplified to reach voltage amplitudes of several hundred volts in order to supply the piezo-electric converter and obtain various stress amplitudes. Both synchronization electronic card and high-voltage amplifier have been specifically developed at the PIMM laboratory. The power of the high-voltage amplifier is about 500 W.

Optimization of the horn and specimen shape and size, using finite-element model computations, ensures that the frequency of the synchronization corresponds exactly to the eigen frequency of the first longitudinal mode of the specimen and the horn. This configuration allows (i) an input voltage signal whose frequency is close to the vibration frequency of the piezo-electric system and (ii) 42 X-ray pulses during a single loading period (*i.e.* 49.61 µs).

#### 2.3.2. Synchronization of the X-ray camera and the strain acquisition

For the acquisition of the X-ray diffraction pattern, the X-ray detector is triggered to capture every 42 X-ray pulses. The associated trigger device is composed of a T560 delay board and a low-voltage generator (branch 2 in Fig. 4 ▸) which allow the trigger signal to be reshaped and an integration time (*i.e.* counting time on the detector) of 0.3 µs to be imposed which is sufficiently small to ensure the capture of a unique X-ray pulse [see Fig. 6(*b*) in Section 2.4]. To reconstruct one loading cycle, the imposed delay is changed to capture successively the 42 pulses (between 0 and 49.61 µs with step of 1.1812 µs). In parallel an acquisition board (RedPitaya STEMlab 125-14) with a sampling frequency of 125 Msamples s^−1^ is triggered by the delay generator board to measure the gauge signal corresponding to the associated X-ray pulse (branch 3 in Fig. 4 ▸).

In order to improve the signal-to-noise ratio of the diffraction pattern, an accumulation of measurements for the same detector delay is carried out using a stroboscopic method. Each diffraction pattern is thus the result of an accumulation of 10^6^ to 4 × 10^6^ snapshot images, each accumulating the diffracted photons from a unique X-ray pulse. Once this accumulation is terminated, the detector image is stored and the detector delay is increased by one step of 1.1812 µs. For total strain measurement, the same stroboscopic method is used and 1500 individual measurements are acquired to compute the mean strain presented below.

**Figure 5:**
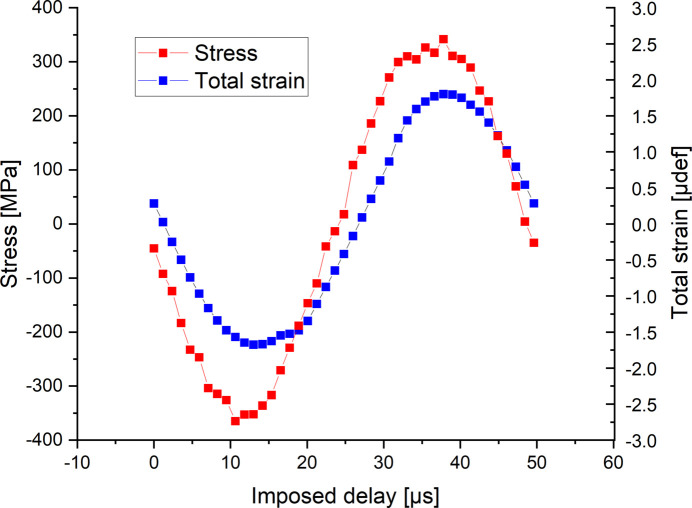
Stress and total strain during one reconstructed cycle.

The accumulation of the diffraction patterns and the gauges measurement is carried out for the 42 X-ray pulses and thus allows the total strain and stress to be obtained during one reconstructed cycle (Fig. 5 ▸).

### 2.4. Temporal characterization of the experimental device

#### 2.4.1. Time shift and jitter induced by the experimental device

**Figure 6:**
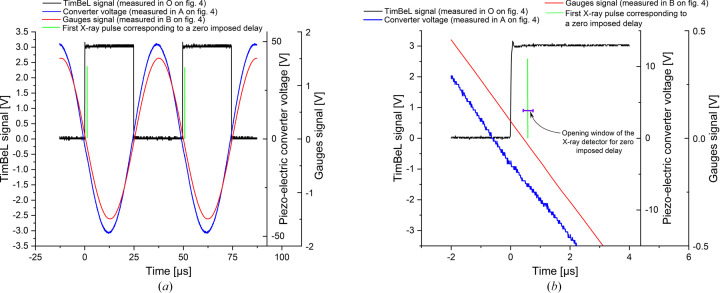
Temporal evolution of the voltage applied to the piezo converter and the gauge signal. (*a*) General view. (*b*) Enlargement.

Temporal resolution is crucial in our experience and several error sources must be identified and quantified. First, the position of the bending magnet of the DiffAbs beamline in the storage ring generates a delay between the trigger given by the TimBeL board (time reference of the measurements), the passage of the bunch at the level of the bending magnet on the front head of the beamline and the arrival of the X-ray pulse on the specimen. This total delay is approximately 680 ns and its variations remains less than a few picoseconds. Moreover, trigger devices, cable lengths, high-voltage amplifier and piezo-electric converter also introduce delays. These delays are inevitable but have the advantage of being nearly constant during all the tests. The sum of these delays can be estimated by calibrating the system during a fatigue test at a very low stress amplitude for which the material only generates a negligible time shift between stress and total strain. Note that this delay remains constant even if the applied stress amplitudes are increased, and only the delay introduced by the material is expected to evolve. For instance, Fig. 6 ▸ gives the time evolution of the voltage applied to the piezo-electric converter after the high-voltage amplification (node A in Fig. 4 ▸), the gauge signal (node B in Fig. 4 ▸) and the synchronization signal delivered by the TimBeL board which gives the time reference (node O in Fig. 4 ▸). These time evolutions are measured in real time using an oscilloscope. In Fig. 6 ▸, the position of the targeted X-ray pulse and the detector opening window for a zero imposed delay, which corresponds to the first targeted X-ray pulse, were added as a vertical green bar and a violet segment, respectively.

In addition to these time shifts, jitter introduced by the triggering and acquisition systems must be considered. It comes from the temporal resolutions and the noise on the signals. These jitters reduce directly the performance of the system and define its limitations. Only two branches of the trigger circuit will impact the time shift between stress and total strain: branch 1 for the synchronization of the fatigue machine and branch 3 for total strain measurement. The trigger branch (branch 2) related to the detector opening window does not introduce time shift and jitter on the stress measurement. As this window is very large (300 ns) compared with the duration of the X-ray pulse (91 ps with a jitter of a few picoseconds), any small jitter in the window position will not affect the capability of the detector to capture the X-ray pulse.

**Figure 7:**
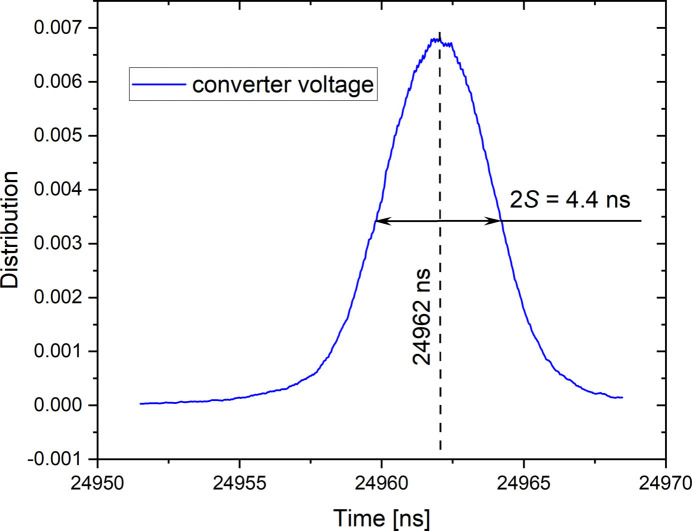
Distribution of the time shift of the converter supply voltage (node A in Fig. 4 ▸). The centre of the distribution corresponds to the delay between the converter voltage signal and the TimBeL rising edge.

Figures 7 ▸ and 8 ▸ show the distribution of the time shifts of the converter supply voltage (node A) and gauge signal (node B) for a supply voltage amplitude of the converter of 50 V corresponding to a stress amplitude of 74 MPa. At this stress amplitude it is expected that the pearlitic steel introduces a negligible time shift between stress and strain. These distributions are characterized by mean values of about 24962 ns and 26116 ns, respectively, for the converter voltage and the gauge signal. The standard deviations of these distributions, representing their jitter, are equal to ±2.2 ns and ±196 ns, respectively. These jitters show that the limitation on the time resolution of our experimental device is mainly due to the gauge measurement (branch 3).

#### 2.4.2. Error in estimating the phase shift of the strain evolution

**Figure 9:**
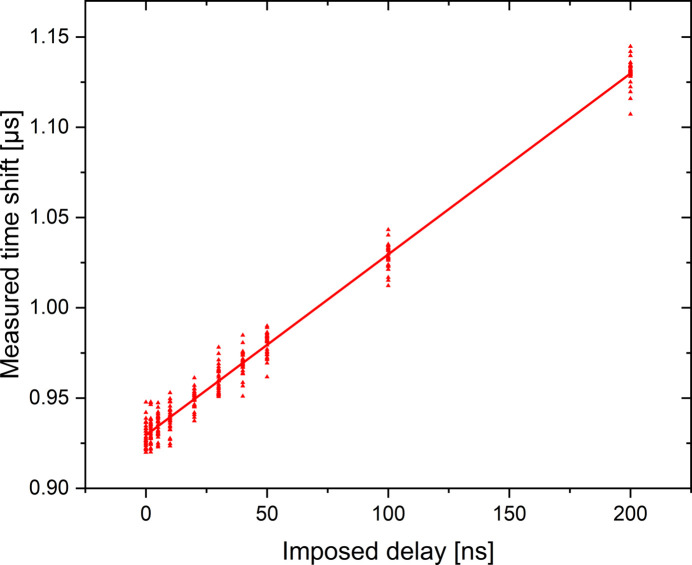
Graphical representation of the acquired and triggering signals. The continuous line represents a linear regression of the whole measurements with a unit slope.

The measured jitter associated with the gauge signal (*2S* = 394 ns) remains too high compared with the expected time shift induced by the material and especially for the low stress amplitude related to the VHCF domain. In order to reduce these jitters, an accumulation of 1500 independent measurements per delay in a loading cycle are performed using the stroboscopic method. The jitter of the mean value is therefore reduced by a factor of about 40. To validate this reduction, an artificial delay is applied by the delay generator board on the triggering of the Redpitaya data acquisition board and the evolution of the gauge signal is obtained for one reconstructed cycle (42 points spaced by 1.1812 µs). The time shift of the strain signal is estimated by a sinusoidal fit on the 42 points. For an imposed delay, the measurement of a time shift is performed 30 times in order to estimate its variability. The results are shown in Fig. 9 ▸ which highlights a global affine evolution of the time shift as a function of the imposed delay. In addition, these measurements allow a standard deviation of the time shift of the strain signal to be estimated as ±7 ns, independently of the imposed delay. This variability gives the temporal resolution of our system on the strain measurement for an accumulation of 1500 measurements.

#### 2.4.3. Uncertainty in estimating the phase shift of the stress evolution

**Figure 10:**
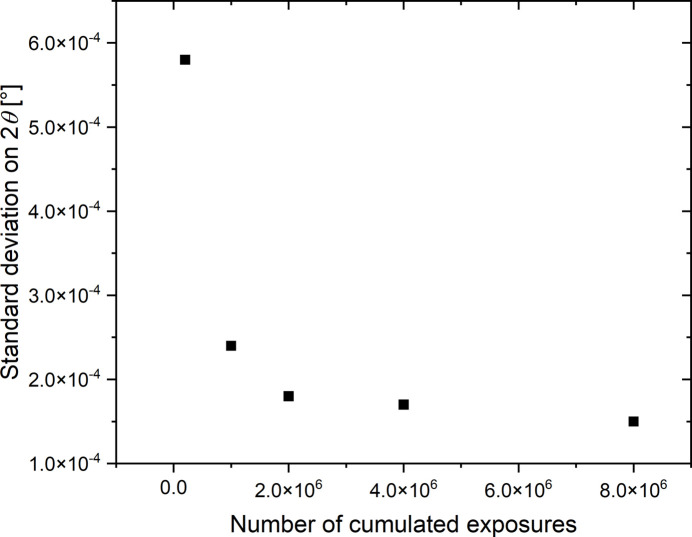
Standard deviation on the peak position estimation according to the number of cumulated exposures. This curve depends on the maximum intensity of the peak which is about 40 kcounts for 10^6^ cumulated exposures.

As mentioned previously, the time shift between the origin of the synchronization given by the TimBeL board and the X-ray pulse arriving on the specimen has a very low variability (about a few picoseconds). The fluctuation on the time shift of the stress signal is thus only related to the estimation of the position of the Bragg peak for the 42 measurement points and to the sinusoidal fit on these 42 values to estimate this time shift. These fluctuations mainly come from the signal-to-noise ratio of the diffraction images, which impacts first on the error on the estimation of the peak position and second on the calculation of the time shift. The accumulation of exposures by the stroboscopic method (∼10^6^ apertures) significantly reduces these errors by directly improving the signal-to-noise ratio of the diffraction images. Moreover, the increase of the displacement amplitude of the Bragg peaks due to the increase in the stress amplitude also reduces the uncertainty on the estimation of their displacement and therefore on the time shift. Figure 10 ▸ gives the standard deviation on the estimate of the peak position when the equivalent number of accumulated exposures is changed. These values are calculated from 50 independent measurements carried out on an unloaded specimen. This figure justifies the fact that the fluctuation on the peak position estimation decreases rapidly with the increase in the number of accumulated exposures. Beyond 2 × 10^6^ exposures, the increase in the number of accumulated exposures improves very little the estimation of the peak position.

**Figure 11:**
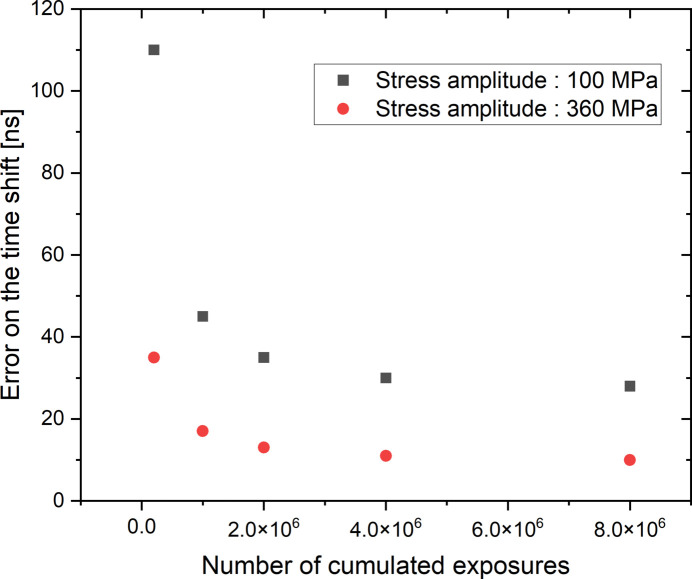
Standard deviation on the time shift estimation according to the number of cumulated exposures.

This fluctuation on the peak position directly influences the estimation of the time shift of the reconstructed stress signal. Figure 11 ▸ represents the standard deviation on the estimation of the time shift of the stress with the reference time given by the TimBeL board. This evolution is obtained by performing 50 cycle reconstructions and thus 50 sinusoidal fits to estimate the time shift jitter. Two stress levels were applied: 100 MPa corresponding to the lower limit of the stress amplitudes applied in this study, and 360 MPa. The general tendency is close to that found for the standard deviation on the peak position. It can also be noted that the increase in stress amplitude leads to a decrease in the standard deviation on the time shift since the amplitudes of displacement of the peak are larger.

## 3. Results and discussion

**Figure 12:**
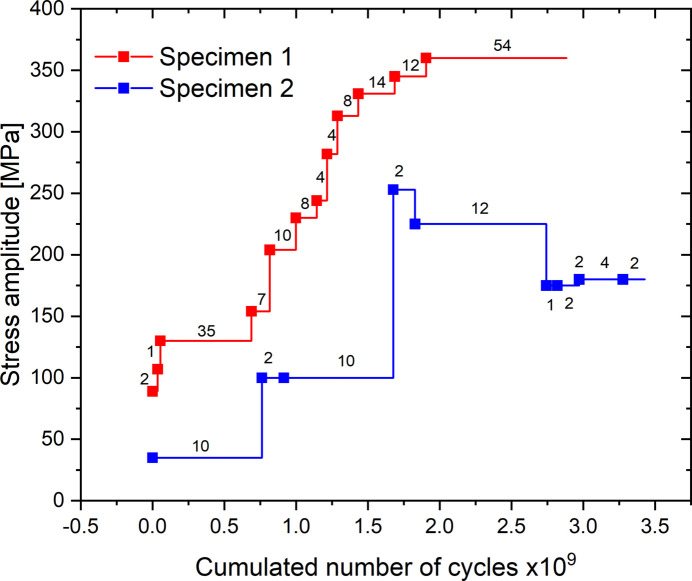
Loading history of the two C70 steel specimens. For each stress amplitude the number of reconstructed cycles is specified on the graph. Each reconstructed cycle is used to estimate a time shift value between stress and strain.

**Figure 13:**
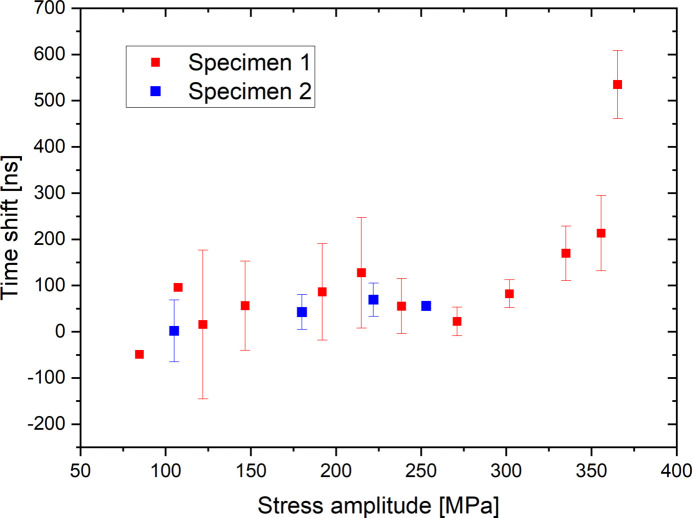
Stress amplitude effect on the evolution of the time shift between stress and total strain for the two specimens. Points with no error bar correspond to a single measurement.

Two C70 steel specimens were stressed at several stress levels (specimen 1 and 2). Figure 12 ▸ gives the loading history of each of these two specimens. For each stress amplitude the number of reconstructed cycles is specified on the graph. Each reconstructed cycle is used to estimate a time shift value between stress and strain. The stress amplitude is thus increased after the reconstruction of several cycles. A single increasing ramp is conducted per specimen. Figure 13 ▸ represents the evolution of this time shift for specimens 1 and 2 as a function of the stress amplitude. Here, we use two accumulated exposure times, 0.5 s for specimen 1 and 2 s for specimen 2, corresponding to accumulations of 10^6^ and 4 × 10^6^ measurements, respectively. The boxes and error bars represent, respectively, the mean value and the standard deviation on the data for the reconstructed cycles carried out at the same stress amplitude. This figure shows first that the results obtained for specimens 1 and 2 are equivalent even if the stress amplitude range for specimen 2 is lower than for specimen 1. In addition, the increase in the stress amplitude also leads to a detectable increase in the time shift.

**Figure 14:**
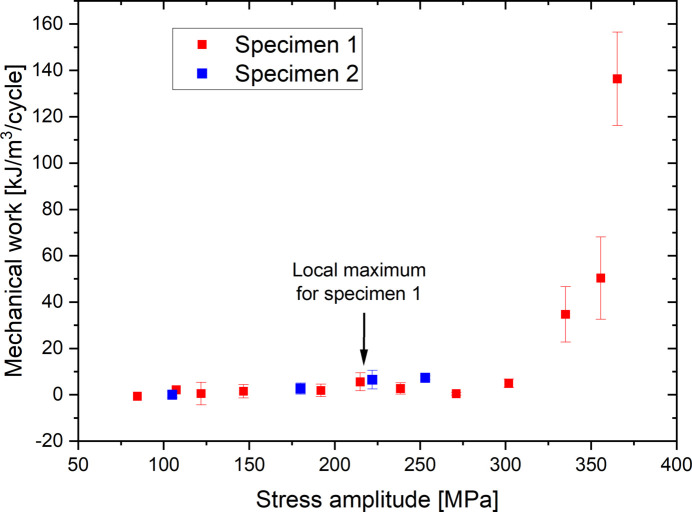
Stress amplitude effect on the evolution of the mechanical work supplied to the material. Points with no error bar correspond to a single measurement.

From this time shift between stress and strain and the respective amplitudes of the stress and strain signals, it is possible to calculate the mechanical work supplied to the material with equation (1). Figure 14 ▸ represents the evolution of the mechanical work according to the stress amplitude for the two specimens. The boxes and the error bars represent, respectively, the mean value and the standard deviation on the work calculated for all the reconstructed cycles at the same stress amplitude. For low stress amplitudes, the mechanical work is very low compared with the fluctuations and is difficult to quantify. The increase in stress amplitude causes an increase in mechanical work which becomes very large for high stress amplitudes greater than 325 MPa. In the case of specimen 1, a small local maximum of the mechanical work can also be distinguished from the noise for stress amplitudes between 200 MPa and 250 MPa. This maximum cannot be highlighted on specimen 2 because the amplitudes of applied stress remain too low. This local maximum could be explained by the formation of dislocation structures which require more mechanical energy to form, but, once these structures are created, the energy supplied to the material decreases.

**Figure 15:**
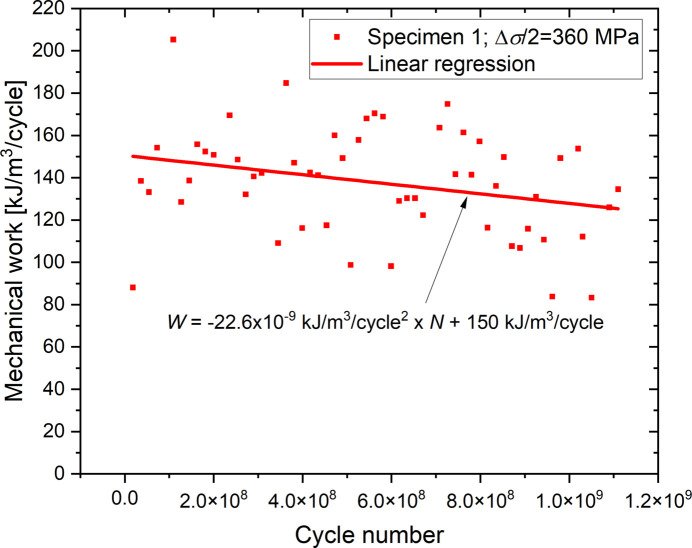
Effect of the number of cycles on the evolution of the mechanical work supplied to the material.

For a stress amplitude of about 360 MPa (specimen 1), 54 cycles were reconstructed covering more than one billion cycles. It is then possible to study the evolution of the mechanical work according to the number of applied cycles. Figure 15 ▸ represents this evolution. Even if the data are quite noisy, it is possible to detect a tendency of decrease in mechanical work as a function of the number of cycles and this also in the domain of the VHCF (number of cycles greater than 10 million). A linear regression can also quantify a decrease of 2.26 × 10^−8^ kJ m^−3^ cycle^−2^.

## 4. Conclusion

The study presented in this paper concerns the development of an original experimental technique to measure the mechanical work supplied during one reconstructed cycle to a metallic material loaded at high frequency (20 kHz) with an ultrasonic fatigue machine. To estimate the mechanical work, it is necessary to simultaneously measure the total strain and the stress with a temporal resolution of about a few tens of nanoseconds. To measure the total strain, we used a strain gauge. For stress estimation a time-resolved X-ray diffraction technique using a pulsed X-ray source was developed. The ultrasonic fatigue machine, the strain and the stress measurement devices are controlled by a synchronization system. The time resolution of our system has been characterized experimentally in order to verify that it is sufficient for a good estimation of mechanical work. The results conducted on a C70 pearlitic steel showed a local maximum of mechanical work for stress amplitudes between 200 MPa and 250 MPa and a significant increase for amplitudes close to the fatigue limit. In addition, for amplitudes close to the fatigue limit, it was also possible to detect and qualify a decrease in mechanical work with the number of cycles and this for more than one billion cycles.

## Figures and Tables

**Figure 8:**
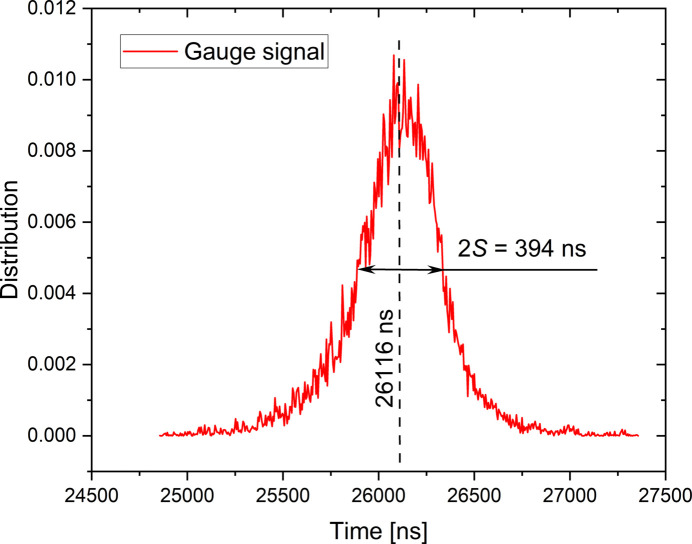
Distribution of the time shift of the gauge signal (node B in Fig. 4 ▸). The centre of the distribution corresponds to the delay between the strain gauge signal and the rising edge of the TimBeL.
